# Ketogenic diet does not promote triple-negative and luminal mammary tumor growth and metastasis in experimental mice

**DOI:** 10.1007/s10585-023-10249-z

**Published:** 2023-12-09

**Authors:** Meret Grube, Arno Dimmler, Anja Schmaus, Rafael Saup, Tabea Wagner, Boyan K. Garvalov, Jonathan P. Sleeman, Wilko Thiele

**Affiliations:** 1 Department of Microvascular Biology and Pathobiology, European Center for Angioscience (ECAS), Medical Faculty Mannheim, University of Heidelberg, TRIDOMUS-Gebäude Haus C, Ludolf-Krehl-Str. 13 - 17, D- 68167 Mannheim, Germany; 2 Institute of Pathology, Vincentius Kliniken Karlsruhe, Karlsruhe, Germany; 3 Institute for Biological and Chemical Systems, Karlsruhe Institute of Technology (KIT), Campus North, Karlsruhe, Germany

**Keywords:** Ketogenic diet, Ketosis, Metastasis, Primary tumor growth, Breast cancer

## Abstract

Ketogenic diets (KDs) can improve the well-being and quality of life of breast cancer patients. However, data on the effects of KDs on mammary tumors are inconclusive, and the influence of KDs on metastasis in general remains to be investigated. We therefore assessed the impact of a KD on growth and metastasis of triple negative murine 4T1 mammary tumors, and on the progression of luminal breast tumors in an autochthonous MMTV-PyMT mouse model. We found that KD did not influence the metastasis of 4T1 and MMTV-PyMT mammary tumors, but impaired 4T1 tumor cell proliferation in vivo, and also temporarily reduced 4T1 primary tumor growth. Notably, the ketogenic ratio (the mass of dietary fat in relation to the mass of dietary carbohydrates and protein) that is needed to induce robust ketosis was twice as high in mice as compared to humans. Surprisingly, only female but not male mice responded to KD with a sustained increase in blood β-hydroxybutyrate levels. Together, our data show that ketosis does not foster primary tumor growth and metastasis, suggesting that KDs can be safely applied in the context of luminal breast cancer, and may even be advantageous for patients with triple negative tumors. Furthermore, our data indicate that when performing experiments with KDs in mice, the ketogenic ratio needed to induce ketosis must be verified, and the sex of the mice should also be taken into account.

**Supplementary Information:**

The online version contains supplementary material available at 10.1007/s10585-023-10249-z.

## Introduction

 Ketogenic diets (KDs) are nutritional therapies that mimic specific aspects of a starvation response. KDs are characterized by a defined ratio of the mass of dietary fats in relation to the mass of dietary carbohydrates and proteins. In the clinic, the ketogenic ratio is adjusted individually for patients with the aim of achieving a state of ketosis that, according to medical guideline, is defined by sustained blood β-hydroxybutyrate (BHB) concentrations of between 2 and 5 mM [1]. In humans, this requirement can typically be fulfilled with a ketogenic ratio of 4:1.

The fat components of KDs are metabolized in the liver, resulting in the production of the three ketone bodies, BHB, acetoacetic acid and acetone. While acetone is partially converted to methylglyoxal, and subsequently catabolized via lactate and pyruvate [2, 3], BHB and acetic acid are released into the circulation, taken up by extrahepatic target cells, and transported to the mitochondria, where they are converted into acetyl-CoA, which then enters the citric cycle to produce ATP.

Mainly known for their successful application in drug-resistant epilepsy patients (reviewed in [4]), KDs can also positively influence body composition of cancer patients [5–8], and improve the well-being and quality of life of patients suffering from different kinds of solid tumors [6, 9–11], including breast cancer [6, 7, 10]. It may therefore make sense to offer KDs to tumor patients as adjunct therapies. However, although several studies with human cancer patients suggest that KDs may also have anti-tumor effects, the significance of these studies is limited by the fact that most studies describing effects of KD on cancer patients are either only case reports or cohort studies that show heterogeneity and/or include only small numbers of patients (reviewed in [12–14]). In the context of breast cancer, preclinical studies employing murine models showed that elevated BHB and lactate levels promoted primary mammary tumor growth and metastasis in experimental mice [15, 16]. In contrast, in studies in which mammary tumor-bearing mice were maintained on KDs, primary tumor growth was at least temporarily reduced [17–21]. However, the impact of the KDs on metastasis was not examined in all of these studies. Where metastasis was assessed, the experimental design and analysis do not allow a conclusive interpretation of the results [19, 20, 22].

In view of these observations, we examined the influence of ketosis on the growth and metastasis of breast tumors in experimental mice. Our results show that ketosis did not affect the metastasis of triple negative 4T1 and luminal MMTV-PyMT mammary tumors in vivo, and only had temporary effects on 4T1 primary tumor growth, suggesting that KDs can be safely applied in the context of luminal and triple negative breast cancer. In addition, our data indicate that the ketogenic ratio required to establish ketosis in humans is insufficient in mice, and a ketogenic ratio of at least 8:1 is required in mice to establish a sustained ketosis with blood BHB concentrations above 2 mM. Moreover, we also found that only female but not male mice responded to KDs with a sustained increase in blood BHB levels, even when a KD with a ketogenic ratio of 10:1 was applied. These findings suggest that dietary regimens used in patients cannot be directly transferred to experimental mice, but have to be adapted to the model organism accordingly. Furthermore, when KDs are fed to mice, the sex of the animals also has to be taken into account.

## Methods

### Tissue culture

4T1 cells were cultivated in DMEM supplemented with 10% FCS and 1% penicillin-streptomycin.

### Experimental mice and genotyping

All animal experiments were approved by the local regulatory authorities (License Numbers: AZ 35-9185.81/G-43/19 and AZ 35-9185.81/G-122/21), and were performed according to German legal requirements. 

BALB/c mice were purchased from Envigo (Venray, Netherlands).

FVB/N-Tg(MMTV-PyMT)634Mul/J (“MMTV-PyMT”) mice express the mammary tumor virus-polyoma middle T (PyMT)-oncoprotein under the control of the mouse mammary tumor virus LTR (MMTV LTR), restricting PyMT expression to the mammary epithelium [23, 24]. MMTV-PyMT mice were bred and maintained on an FVB/N background. Genotyping was performed by PCR using genomic DNA as a template. “Plg-in2-3’” (tgt ggg ctct aaa gat gga act cc) and “Plg-ex2-5’” (gac aag ggg act cgc tgg atg gct a) primers that detect the plasminogen gene that serves as a positive control were used in PCR reactions (95°C, 45 s; 60°C, 45 s; 72°C, 45 s; 35 cycles; 72°C, 10 min), resulting in an amplicon of 268 nucleotides. Detection of PyMT, indicating a transgenic allele, was achieved by using “PyMT3p” (cgg cgg agc gag gaa ctg agg aga g) and “PyMT4m” (tca gaa gac tcg gca gtc tta ggc g) primers in PCR reactions creating an amplicon of 160 nucleotides with the following conditions: 95°C, 45 s; 60°C, 45 s; 72°C, 45 s; 35 cycles; 72°C, 10 min.

Mice were kept in groups of four in type III and type IIL polysulfon IVC cages (Tecniplast, Hohenpeißenberg, Germany) containing soft bedding (Mini Chips LTE E-003 L-10 ABEDD B, Ssniff GmbH, Soest, Germany). Mice were also provided with nesting material (Pura Cocoons Soft Cotton Cylinder, Code 211–1240 HV/MG/0822, Labodia, Switzerland), a mouse house and tubes (Play Tunnels: CS3B01 213–1041) made of cardboard and wooden sticks (Nagehölzer, H0234-NGS, Ssniff and Pura Sticks, Code 213–1001, Labodia, Switzerland) for gnawing. The cages were changed exclusively under laminar flow conditions. Rat/mouse maintenance complete feed (Ssniff; composition see below) and tap water were provided *ad libitum*. The specific pathogen-free housing was kept at 20–22°C and 45–60% humidity on a 06:00–18:00 h light cycle.

Routine health monitoring was carried out in accordance with FELASA recommendations [25]. Parasitological tests were performed in the laboratory of the Core Facility Preclinical Models, bacteriological tests in the Institute of Microbiology, Klinikum Mannheim and serological and PCR tests at BioDoc, Labor für Biomedizinische Diagnostik, Hannover and GIM (Gesellschaft für innovative Mikroökologie mbH, Michendorf, Germany).

### KD and standard diet formulations

Both KD and standard diet were purchased from Ssniff. Standard diet contained 19.0% crude protein, 3.3% crude fat, 5.0% crude fiber, 6.4% crude ash, 25.9% starch, 5.4% sugar and 54.6% N free extracts (ketogenic ratio: 0.055:1). The ingredients, contents, and fatty acid fractions of KD with the different ketogenic ratios provided by the manufacturer are summarized in Table 1.

**Table 1:** Composition of KDs with the different ketogenic ratios used in this study

	3:1	4:1	5:1	6:1	8:1	10:1
**Ingredient [%]**						
Casein	18.90	15.00	12.90	12.00	9.60	8.00
Maltodextrin	4.00	3.10	2.00	0.60	0.30	0.15
Sucrose	0.50	0.50	0.50	0.50	0.20	0.05
Cellulose powder	4.00	4.00	4.00	4.00	3.15	2.20
l-Cystine	0.30	0.25	0.20	0.20	0.15	0.10
Vitamin premixture	1.00	1.00	1.00	1.00	1.00	1.00
Mineral premix	5.00	5.00	5.00	5.00	5.00	5.00
Choline	0.20	0.20	0.20	0.20	0.20	0.20
Corn oil	7.00	7.50	7.70	8.00	8.40	8.70
Butter fat, dehydrated	10.90	11.70	12.30	12.50	13.10	13.60
Pork lard	48.20	51.80	54.20	56.00	58.90	61.00
**Proximate contents [%]**						
Crude protein	16.7	13.3	11.4	10.7	8.6	7.1
Crude fat	66.1	71.0	74.2	76.5	80.3	83.2
Crude fiber	4.0	4.0	4.0	4.0	3.1	2.2
Crude ash	4.5	4.4	4.4	4.4	4.3	4.3
Sugar (sucrose)	1.5	1.5	1.5	1.5	1.2	1.1
Dextrin	4.0	3.0	2.0	0.6	0.3	0.2
**Fatty acids, % in the diet**						
C 4:0	0.42	0.44	0.47	0.48	0.50	0.52
C 6:0	0.27	0.29	0.31	0.32	0.33	0.34
C 8:0	0.15	0.16	0.17	0.17	0.18	0.19
C 10:0	0.38	0.40	0.42	0.43	0.45	0.47
C 12:0	0.47	0.50	0.53	0.54	0.56	0.59
C 14:0	1.80	1.93	2.02	2.07	2.17	2.25
C 16:0	15.31	16.44	17.20	17.72	18.62	19.28
C 18:0	7.69	8.26	8.65	8.92	9.37	9.71
C 20:0	0.19	0.21	0.22	0.22	0.23	0.24
C 16:1	1.63	1.75	1.83	1.89	1.99	2.06
C 18:1	24.68	26.51	27.70	28.59	30.05	31.13
C 18:2	8.48	9.10	9.44	9.78	10.27	10. 64
C 18:3	0.60	0.65	0.68	0.70	0.73	0.76
C 20:4	0.82	0.88	0.92	0.95	1.00	1.04

### Assessment of the effect of different ketogenic ratios

BALB/c and MMTV-PyMT mice (8 weeks old) were fed standard diet or KD (both Ssniff) with ketogenic ratios of 3:1, 4:1, 5:1, 6:1, 8:1 and 10:1 (BALB/c ) or 6:1, 8:1 and 10:1 (MMTV-PyMT) *ad libitum*. The animals were weighed, and blood was taken from the tail vein twice per week for 8 weeks for BALB/c mice or, for the MMTV-PyMT mice, until an individual tumor reached the legal limit of 1.5 cm in one dimension or when the sum of the largest diameters of several tumors reached the legal limit of 3 cm. BHB and glucose concentrations were measured using an Abbot Freestyle Precision Neo H combined glucose/blood ketone meter (Abbott Diagnostics, Wiesbaden, Germany) with the appropriate glucose or β-ketone strips (Abbott Diagnostics), respectively.

### Spontaneous metastasis assay

A KD with a ketogenic ratio of 8:1 was initiated in 8-week-old BALB/c mice *ad libitum*. Animals in the control group were maintained on standard chow. The respective diets were maintained throughout the duration of the experiment. Syngeneic 4T1 cells (1 × 10^6^ each animal) were injected orthotopically into the mammary fat pads of the mice of both KD and control groups 10 days after the initiation of KD. The animals were weighed, and blood was taken from the tail vein twice per week. BHB and glucose concentrations were measured using a combined glucose/blood ketone meter with the appropriate glucose or β-ketone strips (Abbott Diagnostics). Tumor size was measured with a micrometer caliper. Once a tumor reached the maximum approved limit of 2 cm in one dimension, or a mouse reached a humane end point, the animal was killed, serum was obtained, and an autopsy was performed. The volume of the draining ipsilateral axillary lymph nodes was assessed as a measure of lymph node metastasis. Contralateral lymph nodes served as a control. The lung was examined for the occurrence of metastases, and nodules were counted under a stereo microscope. The tumors and surrounding tissue were isolated, formalin-fixed and embedded in paraffin for histological studies.

### Experimental metastasis assay

Maintenance on a KD with a ketogenic ratio of 8:1 was initiated in 8-week-old BALB/c mice *ad libitum*. Animals of the control group were maintained on the standard diet. The respective diets were maintained for the duration of the experiment. Syngeneic 4T1 cells (2.5 × 10^4^ each animal) were injected into the tail vein of the mice of both KD and control groups 10 days after the initiation of the KD. The animals were weighed regularly, and blood was taken from the tail vein twice per week. BHB and glucose concentrations were measured using a combined glucose/blood ketone meter with the appropriate glucose or β-ketone strips (Abbott Diagnostics). The animals were killed on day 21 post tumor cell injection, and serum was obtained. An autopsy was performed, and the lung was examined for the occurrence of metastases. Metastatic nodules were counted under a stereo microscope.

### Autochthonous model

Maintenance on a KD with a ketogenic ratio of 8:1 was initiated in 8 week old MMTV-PyMT mice *ad libitum*. Animals in the control group were maintained on the standard diet. The respective diets were maintained for the duration of the experiment. The animals were weighed, and blood was taken from the tail vein twice per week. BHB and glucose concentrations were measured using an Abbot Freestyle Precision Neo H with the appropriate glucose or β-ketone strips (Abbott Diagnostics). Tumor size was measured with a micrometer caliper. Once an individual tumor reached the legal limit of 1.5 cm in one dimension or when the sum of the largest diameters of several tumors reached the legal limit of 3 cm, or a mouse reached a humane end point, the animal was killed, serum was obtained, and an autopsy was performed. The number of primary tumors was counted, and the volume of the draining ipsilateral axillary lymph nodes was assessed as a measure of lymph node metastasis. Contralateral lymph nodes served as a control. The lung was examined for the occurrence of metastases, and nodules were counted under a stereo microscope. The tumors and surrounding tissue were isolated, formalin-fixed and embedded in paraffin for histological studies.

### Histology and immunohistochemistry

 Pieces of tumor were formalin-fixed and embedded in paraffin wax. Paraffin wax-embedded section (5 μm thick) were boiled in citric acid-based buffer (Dako, Hamburg, Germany), pH 6.1 in a steam cooker for 30 min to retrieve antigens, then blocked with 10% goat serum in PBS, and incubated with primary antibodies directed against Ki-67 (Abcam, Cambridge, UK) or Hcar2 (Novus Biologicals, Centennial, CO) at 1 µg/ml or 0.5 µg/ml, respectively, overnight at 4 °C. Binding of the primary antibodies was visualized using ABC-HRP reagent with Nova Red (Vector Laboratories, Newark, CA). Cell nuclei were counterstained with hematoxylin. Specimens were examined and imaged on an AxioImager Z1 microscope equipped with an AxioCam HRc camera (Carl Zeiss Microscopy, Jena, Germany). The proliferative index (percentage of Ki-67 positive cells) and the Hcar2 positive area [%] as well as the staining intensity (Nova Red signal intensity in the Hcar2-positive area) were quantified with Fiji/ImageJ (PMID 22,743,772). For this, three representative images of each stained tumor section were analyzed, and their mean values were assessed. Mean values for each group (standard diet or KD group) were then calculated.

### Statistics

Testing for statistical significance was performed either by 2-tailed unpaired *t* tests, a mixed-effects model with Šídák’s multiple comparisons test (tumor growth) or Fisher´s Exact Tests (proportion of animals with pulmonary metastasis).

## Results

### A ketogenic ratio of 8:1 is necessary to induce sustained ketosis in BALB/c mice

In the clinic, KDs are usually initiated with a ketogenic ratio of 4:1 that is then further adjusted for each individual patient to achieve a blood BHB concentration between 2 and 5 mM, which define the recommended state of ketosis [1]. To establish the optimal ketogenic ratio that would induce sustained ketosis in BALB/c mice, a strain that is syngeneic for 4T1 triple-negative breast cancer cells that we intended to use for spontaneous and experimental metastasis assays, we fed BALB/c mice *ad libitum* (N = 4; 2 males and 2 females per group) with standard diet or with KDs containing ketogenic ratios of 3:1, 4:1, 5:1, 6:1, 8:1 and 10:1. Blood BHB, blood glucose and the weight of the animals was monitored for 8 weeks.

**Fig. 1:**
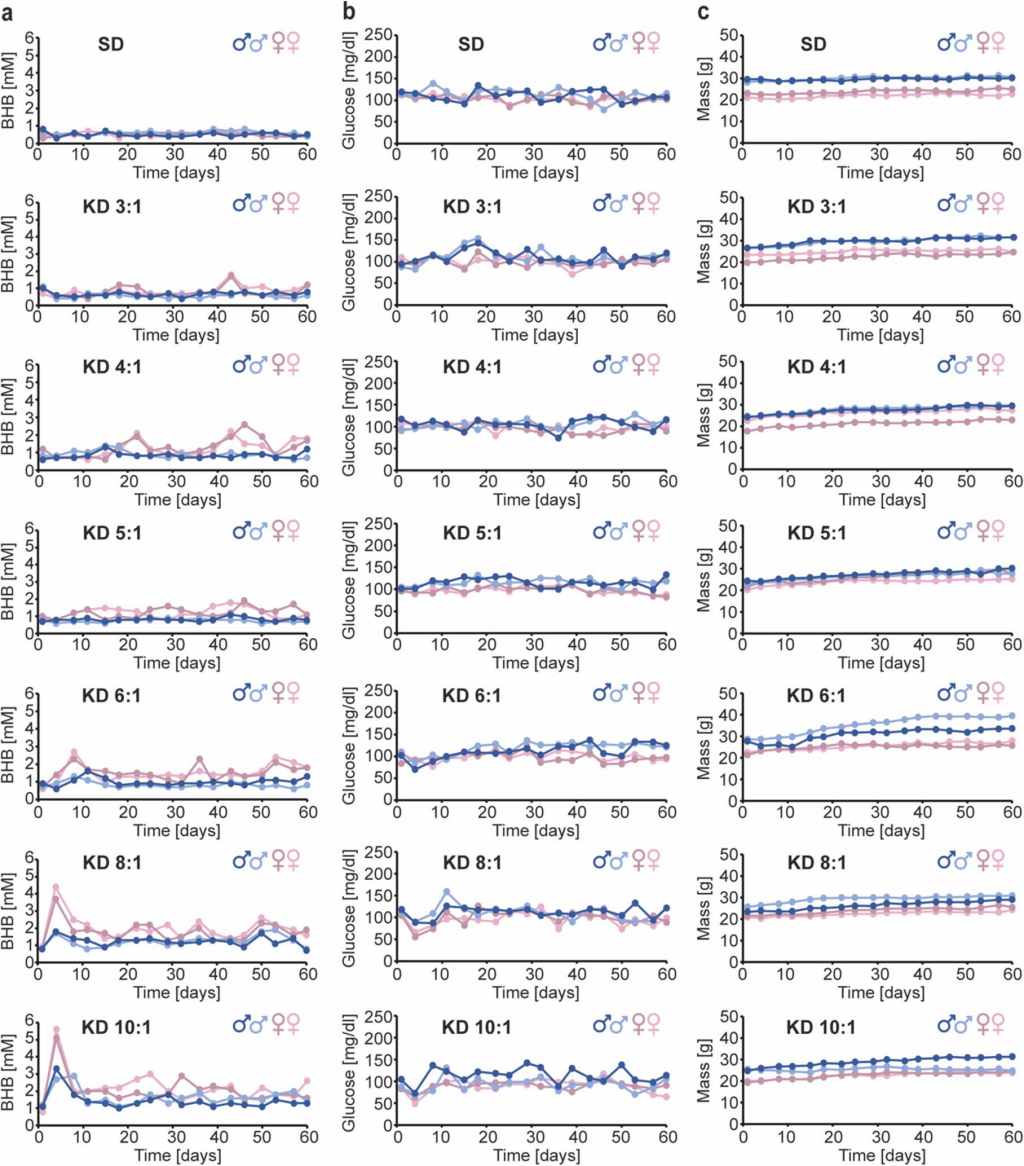
Sustained ketosis can be induced by KDs containing ketogenic ratios of 8:1 and 10:1 in female but not in male BALB/c mice. BALB/c mice (N = 4; 2 males and 2 females per group) were fed with standard diet (SD) or ketogenic diet (KD) with ketogenic ratios of 3:1, 4:1, 5:1, 6:1, 8:1 and 10:1 *ad libitum*. Blood BHB (**a**), blood glucose (**b**) and the weight of the animals (**c**) were monitored regularly for 8 weeks. The lines represent the values for the four individual animals

Only KD ratios of 8:1 and 10:1 induced elevated and sustained ketosis (Fig. 1a). With both of these ketogenic ratios, BHB levels peaked 4 days after KD initiation, reaching levels of between 4 and 5 mM. BHB levels were reduced at day 8, and had reached a stable state at day 11 post KD initiation, which then remained constant until the end of the observation period. Surprisingly, only female but not male animals responded to the diet and reached stable and sustained blood BHB concentrations above 2 mM (Fig. 1a). There were no significant changes in blood glucose and weight between the experimental groups (Fig. 1b, c).

These findings indicate that the ketogenic ratios that are needed to induce sustained ketosis, defined by blood BHB concentrations above 2 mM, are much higher in mice compared to humans, and that male mice do not respond to KDs with sustained elevated blood BHB levels, even with a ketogenic ratio of 10:1.

### KD only temporarily reduces primary tumor growth and does not affect metastasis in a spontaneous metastasis model of triple-negative breast cancer

KD is characterized by the production of BHB from dietary fats. Since BHB has been reported to promote triple-negative breast tumor growth [15], and the influence of KD on metastasis in general remains to be investigated, we assessed the effect of KD in a spontaneous metastasis assay using triple-negative 4T1 mammary carcinoma cells inoculated into syngeneic BALB/c mice.

For the KD, we chose a ketogenic ratio of 8:1. Our finding that male mice did not respond to KDs with elevated blood BHB concentrations (Fig. 1a) did not limit our experiments, since the use of female mice is the natural choice for breast cancer studies. We injected syngeneic 4T1 triple-negative tumor cells orthotopically into the fourth mammary fat pad 10 days after the initiation of the KD, a timepoint at which the concentrations of BHB in the blood have reached a stable state (Fig. 1a). Furthermore, based on observations from the literature, we reasoned that the effect of the KD would be most pronounced when the diet was initiated before tumor cell inoculation [26]. The KD was maintained throughout the experiment. Blood BHB, glucose, weight of the animals, and tumor growth were monitored, and the animals were killed once the tumors reached the legal limit of 2 cm in one dimension. Post mortem examinations were performed, and primary tumors, the lungs and axillary lymph nodes were removed. The number of metastases on the surface of the lung lobes was counted, and the volume of the axillary lymph nodes was calculated.

**Fig. 2:**
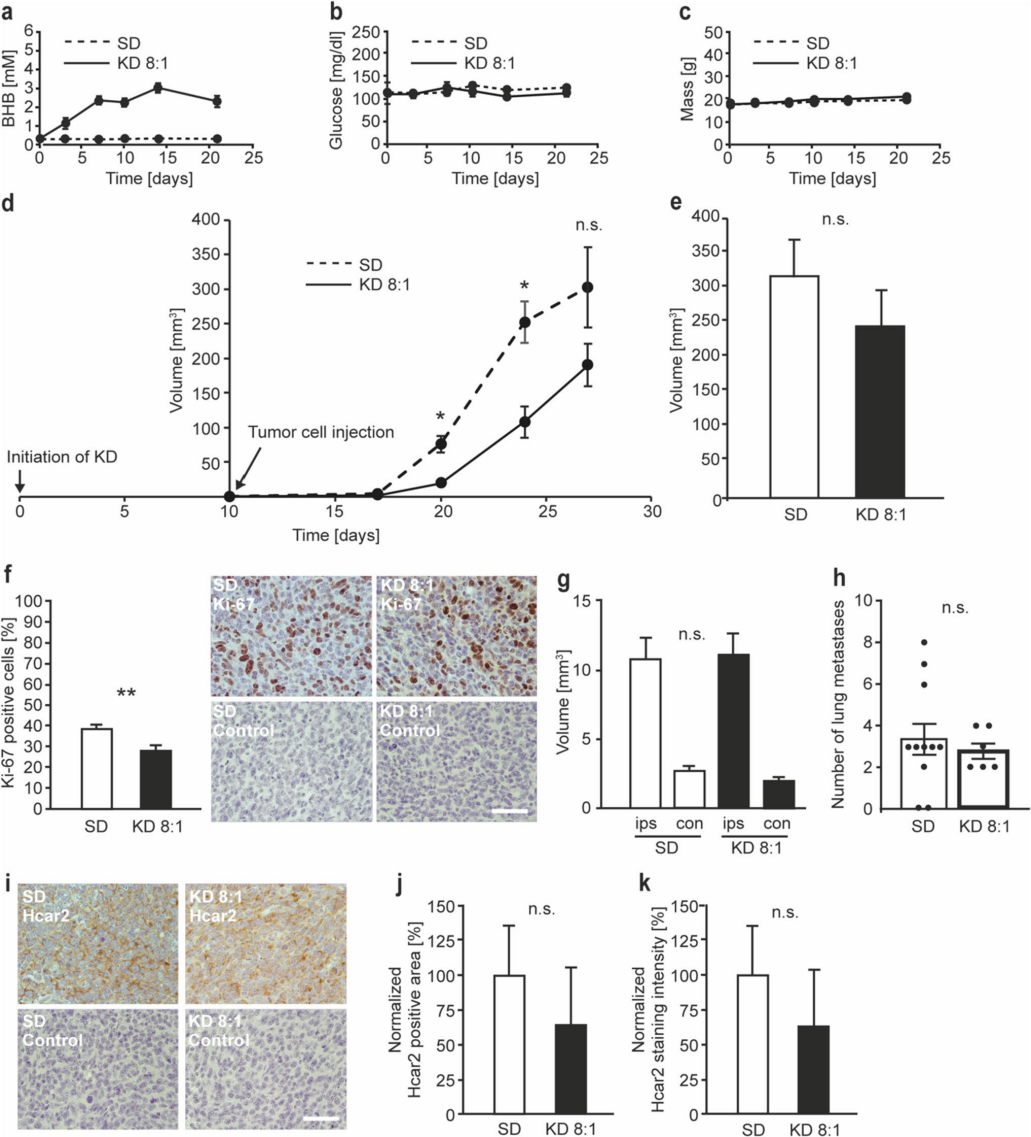
KD temporarily reduces primary tumor growth but does not affect metastasis to lymph nodes and lung in a spontaneous metastasis assay upon orthotopic implantation of 4T1 mammary carcinoma cells. Female BALB/c mice were either fed standard diet (SD; N = 11 animals) or ketogenic diet (KD; N = 6 animals) with a ketogenic ratio of 8:1 *ad libitum*. 4T1 cells (1 × 10^6^ each animal) were injected orthotopically into the mammary fat pads of syngeneic BALB/c mice 10 days after the initiation of KD. (**a**) BHB and (**b**) glucose concentrations were longitudinally measured in the peripheral blood. (**c**) The weight of the animals was determined. (**d**) Tumor growth was monitored regularly, and the animals were killed as soon as the tumors reached the legal limit of 2 cm in one dimension, or when the animals reached a humane endpoint. The final measurement shown was made when the first animal had to be taken out of the experiment for these reasons. Three animals of the SD group were killed on day 27, the remaining animals were killed on day 29. Tumor volume was calculated assuming spherical geometry. The volumes of the primary tumors are plotted against time. (**e**) Final volumes of the primary tumors measured when the animals were killed. (**f**) Paraffin sections of the primary tumors were stained with antibodies specific for Ki-67, and the proliferative index was determined. (**g**) Post mortem examinations were performed, and the dimensions of the ipsilateral tumor-draining axillary lymph nodes (ips) were measured, and their volume calculated, assuming spherical shape. Contralateral lymph nodes (con) served as internal controls. (**h**) The number of superficial lung metastases was counted. (**i**) Histological sections of the primary tumors were stained with antibodies specific for Hcar2. (**j**) The area and (**k**) intensity of the Hcar2 staining was assessed. Error bars represent SE (*p < 0.05; *n.s.* not significant). Scale bars: 50 μm

In the KD group, blood BHB concentrations reached a stable state above 2 mM 8 days post initiation of the KD, and this level was maintained throughout the duration of the experiment (Fig. 2a). Blood glucose was only marginally reduced in the group fed with the KD in comparison with the standard diet group (Fig. 2b), while the weight of the animals was not affected by the KD (Fig. 2c). Tumor growth was statistically significantly reduced in the KD group for the first 2 weeks after tumor cell inoculation, but the difference became less pronounced and was no longer statistically significant at day 27 post tumor cell inoculation and later (Fig. 2d, e). Nevertheless, at the end of the experiment, the proliferative index, determined by Ki-67 staining of the primary tumors was still statistically significantly lower in the KD group (Figs. 2f, S1a). The volume of the axillary lymph nodes did not differ between the KD and the standard diet groups (Fig. 2g), and the number of pulmonary metastatic nodules was not statistically significantly reduced in the KD group (Fig. 2h). Although the proportion of mice developing pulmonary metastases was slightly higher in the KD than in the standard diet group (6/6 animals with metastases vs. 9/11 animals with metastases), the effect was not statistically significant (Fisher’s Exact Test, p = 0.5147). Our data indicate that a KD can suppress the proliferation of 4T1 tumor cells, and can temporarily impair the growth of primary 4T1 tumors.

Since the slight reduction in blood glucose levels was too small to explain the reduction in primary tumor growth, we considered alternative mechanisms. A recent report shows that a KD can substantially reduce colon carcinoma growth via activation of BHB receptor Hcar2 [27]. In order to test whether Hcar2 is present in 4T1 tumors and if the expression of the Hcar2 differs between mice fed KD or standard diet, we stained sections of primary 4T1 tumors with antibodies specific for Hcar2. Hcar2 was ubiquitously expressed in 4T1 tumors (Figs. 2i, S1b). However, although there was a slight tendency towards reduced expression in the KD group, neither the positively-staining area nor the staining intensity was statistically significantly different between the KD and the standard diet groups (Fig. 2j, k).

Together, these data suggest that despite the prominent expression of BHB receptor Hcar2, KD can impair the growth of primary triple-negative breast tumors only temporarily, and has no significant impact on spontaneous metastasis.

### KD does not affect lung metastasis formation in an experimental metastasis model of triple-negative breast cancer

**Fig. 3:**
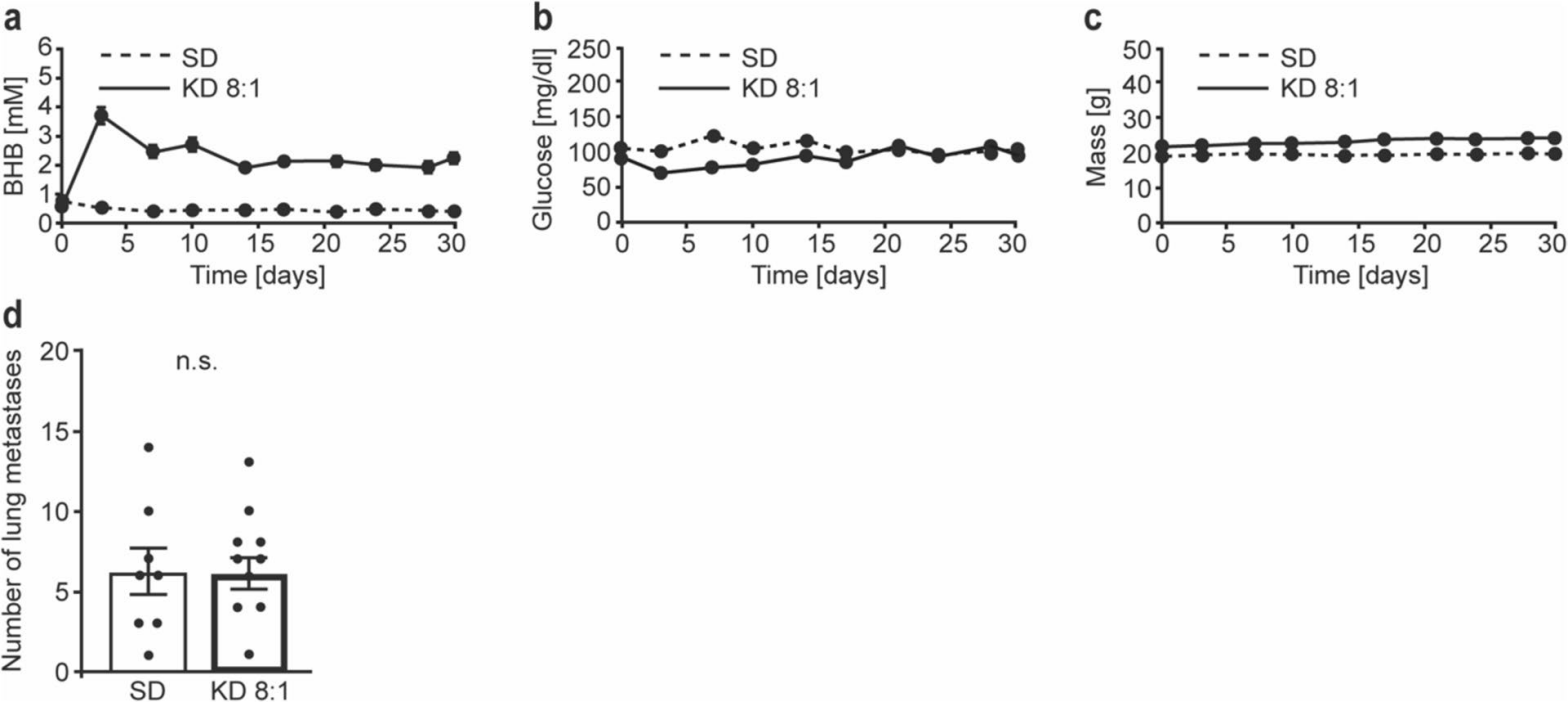
The number of superficial 4T1 lung metastases is not affected by KD compared to standard diet in an experimental metastasis model. Female BALB/c mice were either fed standard diet (SD) or ketogenic diet (KD) with a ketogenic ratio of 8:1 *ad libitum*. 4T1 mammary carcinoma cells (2.5 × 10^4^ each animal) were injected into the tail vein of the mice 10 days after the initiation of KD. (**a**)–(**c**) Blood BHB (**a**), blood glucose concentrations (**b**) and the weight of the animals (**c**) were assessed longitudinally throughout the duration of the experiment. The animals were killed on day 21 post tumor cell injection. (**d**) The lungs were removed and the number of superficial lung metastases was quantified. (SD: N = 8; KD: N = 11) Error bars represent SE (*n.s.* not significant)

Although the number of lung metastases did not significantly differ between mice receiving standard diet or KD (Fig. 2h), the effect of the KD on tumor cell proliferation and primary tumor growth that we observed in the spontaneous metastasis model (Fig. 2d, f) may nevertheless have had an impact on metastasis formation, because metastasis is a function of primary tumor volume in spontaneous metastasis models, and the KD may therefore also have influenced the efficiency of the metastatic process. In order to assess a possible influence of KD on metastasis directly, we therefore performed an experimental metastasis assay in which primary tumor growth is bypassed by the injection of the tumor cells directly into the circulation, resulting in metastatic colonization of the lung. To this end, we injected syngeneic 4T1 triple-negative tumor cells into the tail vein of female BALB/c mice 10 days after the initiation of maintenance on a KD (8:1), or into mice receiving standard diet. Blood BHB and glucose levels as well as the weight of the animals were monitored regularly. The animals were killed 3 weeks after tumor cell injection, the lungs were removed, and the number of surface lung metastases was counted. As before, ketosis was robustly induced by the KD (Fig. 3a). Glucose levels (Fig. 3b) and body weight (Fig. 3c) were not significantly affected by the diet. Notably, the number of lung metastases that developed did not differ between mice fed with the KD compared with mice that received the standard diet (Fig. 3d). Taken together, these results show that a KD does not inhibit, but also does not promote the ability of 4T1 tumor cells to colonize the lungs in experimental metastasis assays, confirming the results obtained with the spontaneous metastasis assay.

### KD does not affect tumor progression in an autochthonous model of luminal breast cancer

In the spontaneous and experimental metastasis assays described above, KD was initiated before the injection of the tumor cells, based on the rationale that the effect of the KD would be more pronounced when the diet was initiated before tumor cell inoculation [26]. However, in the clinical setting, patients with breast cancer would most likely start a KD only after diagnosis. In order to better model the clinical scenario, and also to assess the effect of a KD in an independent mammary carcinoma model, we employed the autochthonous MMTV-PyMT model for luminal breast cancer, and initiated maintenance on a KD when the mice were 8 weeks old. At 8 weeks of age, MMTV-PyMT tumors have progressed to early carcinoma [24], a stage of breast cancer that is often present at diagnosis in human patients.

In contrast to the BALB/c background that was appropriate for the injection of syngeneic 4T1 cells, the MMTV-PyMT mice were maintained on an FVB background. Since the genetic background may influence the response to different ketogenic ratios, we first tested the effect of different ketogenic ratios in female MMTV-PyMT mice. To this end, we assessed blood BHB, blood glucose and the weight of mice (4 animals per group, 8 weeks old) fed with KD of ketogenic ratios 6:1, 8:1 and 10:1 *ad libitum* for up to 35 days.

**Fig. 4:**
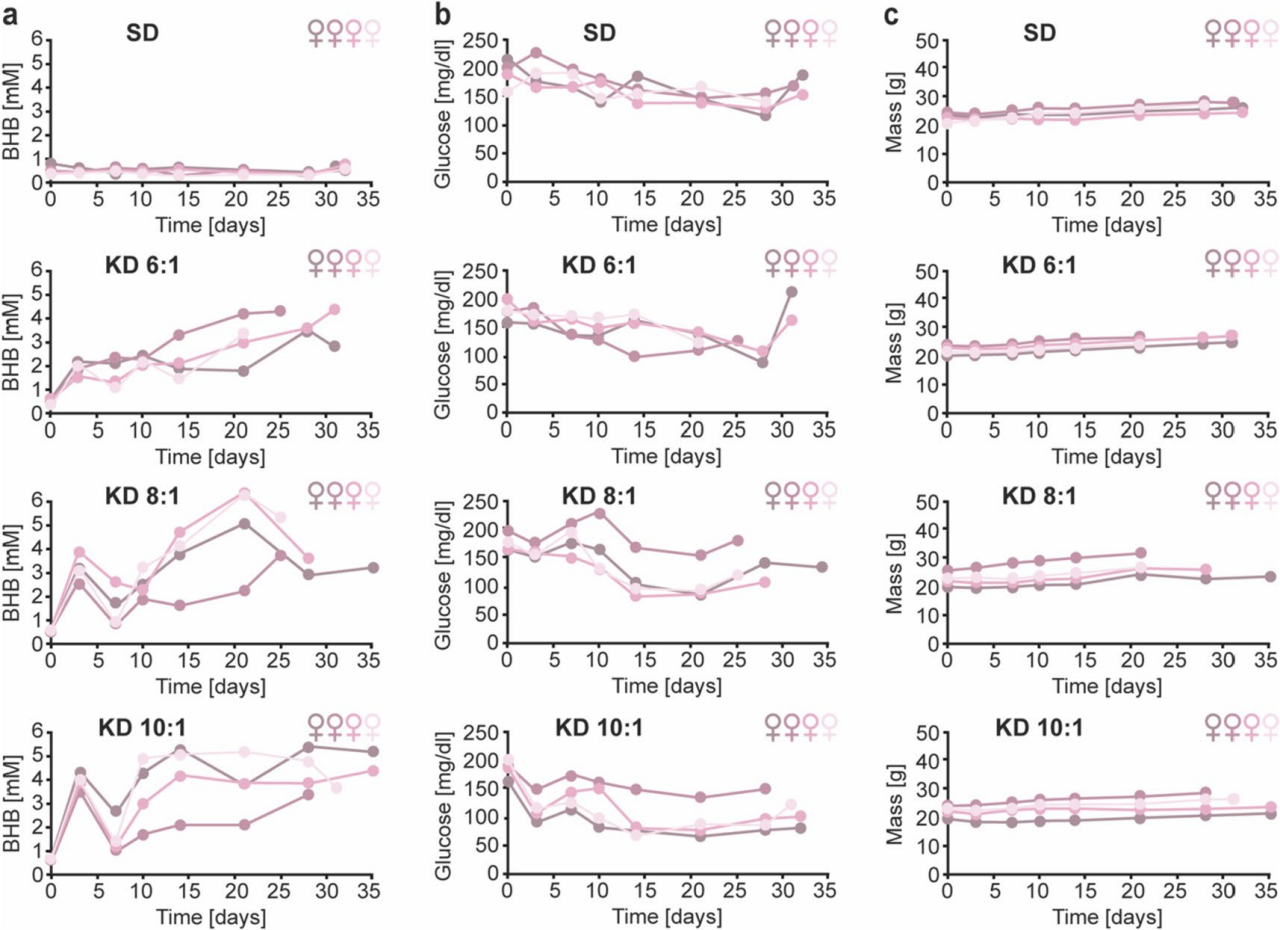
Sustained ketosis can be induced in female MMTV-PyMT mice by feeding them with KDs containing ketogenic ratios of 8:1 and 10:1. MMTV-PyMT mice (N = 4 females per group) aged 8 weeks were fed either standard diet (SD) or ketogenic diet (KD) with ketogenic ratios of 6:1, 8:1 and 10:1 *ad libitum*. Blood BHB (**a**), blood glucose (**b**) and the mass of the animals (**c**) were monitored regularly for up to 35 days. The developing primary tumors were measured regularly, and the animals were killed as soon as an individual tumor reached the legal limit of 1.5 cm in one dimension or when the sum of the largest diameters of several tumors reached the legal limit of 3 cm

In analogy with the results obtained with BALB/c mice (Fig. 1a), ketogenic ratios of 8:1 and 10:1 but not 6:1 induced sustained ketosis with blood BHB concentrations markedly above 2 mM in MMTV-PyMT mice (Fig. 4a). While the weight of the mice did not differ between the individual groups throughout the observation period (Fig. 4c), blood glucose was significantly reduced in MMTV-PyMT mice receiving a 10:1 KD (Fig. 4b).

In view of these observations, we used a ketogenic ratio of 8:1 for the subsequent experiments. Starting at 8 weeks of age, female MMTV-PyMT mice (13 animals per group) were fed with either a KD containing a ketogenic ratio of 8:1 or with standard diet. The feeding regime was then continued for the duration of the experiment. Blood BHB and glucose levels, the weight of the animals as well as the number and growth of the autochthonous tumors were monitored. The animals were killed once an individual tumor reached the legal limit of 1.5 cm in one dimension or when the sum of the largest diameters of several tumors reached the legal limit of 3 cm. Post mortem examinations were performed, during which the primary tumors, the lungs and axillary lymph nodes were removed. The number of metastases on the surface of the lung lobes was counted, and the volume of the axillary lymph nodes was calculated.

**Fig. 5:**
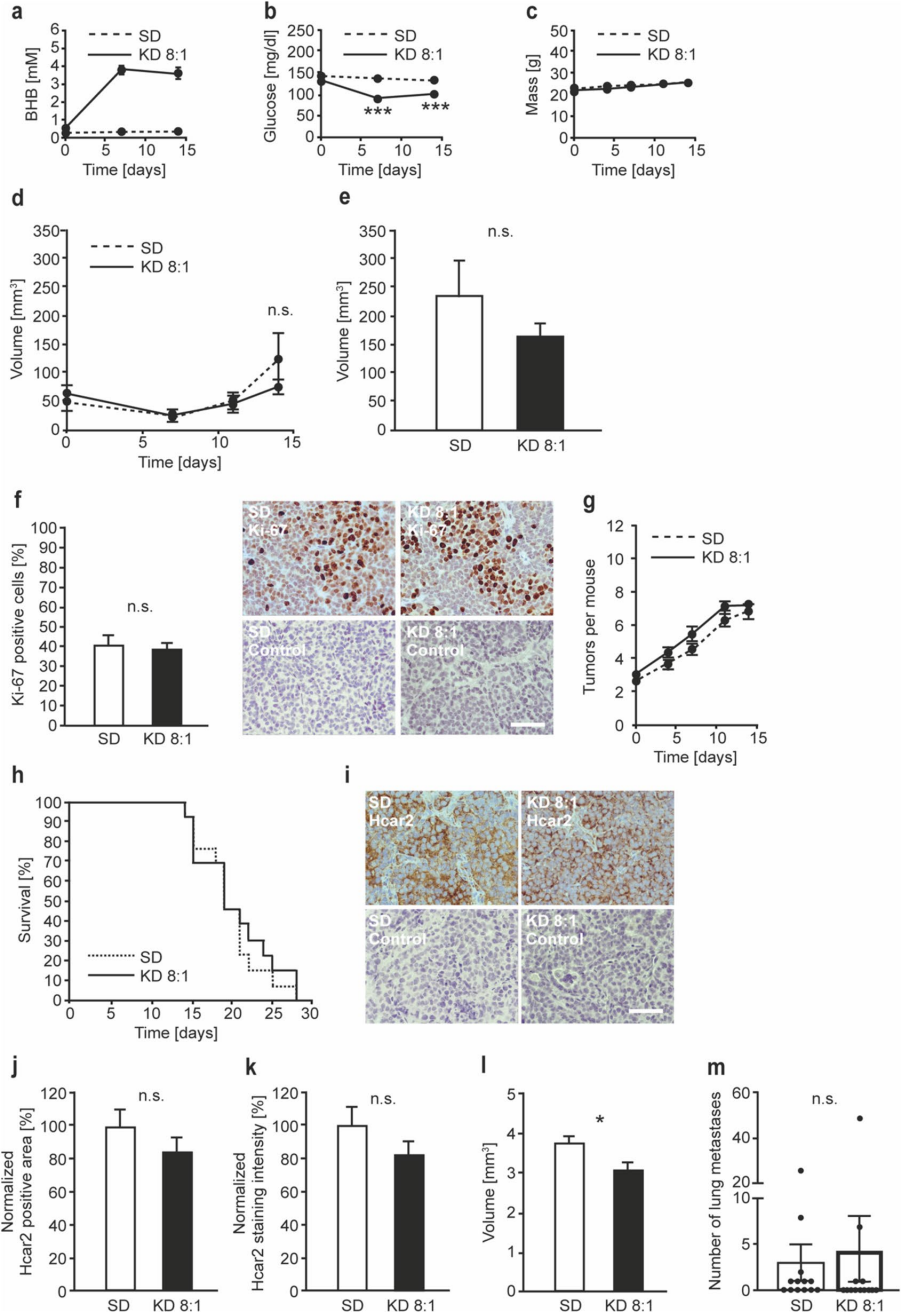
KD does not affect the progression of autochthonous luminal MMTV-PyMT tumors. MMTV-PyMT mice (N = 13 females per group) aged 8 weeks were either fed standard diet (SD) or ketogenic diet (KD) with a ketogenic ratio of 8:1 *ad libitum*. (**a**) BHB and (**b**) glucose concentrations were longitudinally measured in the peripheral blood. (**c**) The weight of the animals was determined. (**d**) Tumor growth was monitored regularly, and the animals were killed as soon as an individual tumor reached the legal limit of 1.5 cm in one dimension, or when the sum of the largest diameters of several tumors reached the legal limit of 3 cm. The final measurement shown was made when the first animal had to be taken out of the experiment. Tumor volume was calculated assuming spherical geometry. The volumes of the primary tumors are plotted against time. (**e**) Final volumes of the primary tumors measured immediately before the animals were killed. (**f**) Paraffin sections of the primary tumors were stained with antibodies specific for Ki-67, and the proliferative index was determined. (**g**) The number of the autochthonous tumors that developed in the individual mice was counted. (**h**) The survival of the mice was assessed. (**i**) Paraffin sections of the primary tumors were stained with antibodies specific for Hcar2. The area (**j**) and intensity (**k**) of the Hcar2 staining was analyzed. (**l**) Post mortem examinations were performed, and the dimensions of the axillary lymph nodes were measured, and their volume calculated, assuming spherical shape. (**m**) The number of superficial lung metastases was counted. (*n.s.* not significant *p < 0.05; ***p < 0.001). Error bars represent SE. Scale bars: 50 μm

As expected, blood BHB concentrations reached a stable state above 3 mM in the KD group 8 days after initiation of the KD (Fig. 5a). However, in contrast to the pilot experiment (Fig. 4b), blood glucose was statistically significantly reduced in the KD group throughout the experiment (Fig. 5b), whereas the weight of the mice did not differ between the KD and the standard diet groups (Fig. 5c). The growth of the primary tumors was not significantly affected by the KD (Fig. 5d). The final tumor volumes were only slightly but not statistically significantly reduced in the KD group (Fig. 5e), which was in accordance with the proliferative index determined by Ki-67 staining of the primary tumors (Figs. 5f, S2a). The number of tumors that the mice developed did not show any statistically significant differences between the KD and standard diet groups (Fig. 5g), and KD also had no influence on the survival of the mice (Fig. 5h). In addition, no difference was found in terms of the stained area (Figs. 5i, j, S2b) and staining intensity (Figs. 5i, k, S2b) of Hcar2 expression in MMTV-PyMT tumors taken from mice fed with the KD or standard diet. Lymph node volumes were slightly, but statistically significantly reduced in the KD group (Fig. 5l). However, analysis of serial sections of the lymph nodes by a trained pathologist revealed that tumor cells were absent from the lymph nodes (data not shown). The number of pulmonary metastatic nodules did not show any statistically significant difference (Fig. 5m). Taken together, these data indicate that in MMTV-PyMT mice, a KD with a ketogenic ratio of 8:1 leads to sustained ketosis, decreased blood glucose levels and decreased lymph node volume, whereas tumor growth and metastasis are not affected.

## Discussion

In this study we performed spontaneous and experimental metastasis assays with 4T1 cells in female mice, and in addition also employed an autochthonous MMTV-PyMT model to investigate the effect of a KD on the growth and metastasis of breast cancer. Under conditions in which the KD induced robust and continuous ketosis, we observed only a temporary reduction on 4T1 primary tumor growth. No effect on the growth of autochthonous MMTV-PyMT tumors was observed, despite a significant reduction in blood glucose levels. In both models, sustained ketosis did not significantly influence metastasis formation of HCAR-2 expressing triple-negative 4T1 and luminal MMTV-PyMT mammary tumors. Taken together, our studies provide pre-clinical support for the notion that KDs should be safe for breast cancer patients.

KDs can improve the well-being and quality of life of patients suffering from breast cancer [6, 7, 10]. However, although breast cancer patients were included in several studies with KDs [6, 28–34], small patient numbers [6, 29, 31] and heterogeneous cohorts [6, 30–34] do not allow any general conclusions to be drawn about the effects of KDs on human breast cancer growth and metastasis. Moreover, although one study with 60 patients (30 patients on standard diet and 30 patients on a KD) with advanced or metastasized breast tumors treated with cytostatic drugs observed prolonged survival of the patients in the KD group, it is not clear if the effect is caused by the diet itself or by the reduction in caloric intake that was associated with the KD, particularly since mean BHB concentrations remained below 1 mM and thus sustained ketosis was not induced [32]. Thus to date, the effect of KDs on breast cancer growth and metastasis in patients has not been definitively assessed.

Preclinical studies have so far also not provided unequivocal data regarding the impact of KDs on mammary tumor progression. Initial studies were based on the injection of BHB into experimental animals, which unexpectedly and alarmingly promoted the growth of xenografted MDA-MB-231 tumors but did not affect metastasis [15]. The growth promoting effects of the injection of BHB on primary tumors were confirmed in an autochthonous mouse model of erbB-2-positive luminal breast cancer [35]. Moreover, overexpression of enzymes involved in ketone body catabolism promoted MDA-MB-231 primary tumor growth and lung metastasis in spontaneous and experimental metastasis assays, respectively [16]. These studies raised concerns about the oncological safety of KDs for breast cancer patients.

The first preclinical breast cancer study in which a KD was actually fed to experimental animals employed a calorically restricted KD with a ketogenic ratio of 6:1, which was initiated after 4T1 tumor cell inoculation into syngeneic BALB/c mice. The KD reduced primary tumor growth, but the impact on metastasis was not investigated [36]. However, studies with calorically restricted KDs combine two effects, and are therefore less informative in terms of the impact of the KD diet itself. Another study with an unrestricted KD with a ketogenic ratio of 4:1 that was initiated 14 days post tumor cell injection and fed to syngeneic BALB/c mice *ad libitum* is therefore perhaps more informative [18]. Here, reduced growth of primary tumors derived from syngeneic triple negative EMT6 mouse breast tumor cell lines was observed, contrasting the findings made with BHB injections. Metastasis was, however, not assessed in this study.

A previous study using the autochthonous MMTV-PyMT model that we employed here used a KD with a ketogenic ratio of 4:1 that was initiated at an age of 6 weeks [19], i.e. 2 weeks earlier than in our experiments. Unlike our results, and despite our finding that a ketogenic ratio of 4:1 is not sufficient to induce sustained ketosis with blood BHB concentrations above 2 mM in MMTV-PyMT mice, the authors reported a significant reduction of tumor growth, but did not observe a difference in lung metastasis [19]. BHB concentrations above 1 mM were reached on day 26 post KD initiation, paralleled by a significant drop in blood glucose levels, and BHB concentrations above 2 mM were observed on day 34. However, a difference in tumor growth between SD and KD fed animals seems already apparent 13 days after KD initiation. It is therefore unclear if ketosis was responsible for the impaired tumor growth. A reduction of MMTV-PyMT primary tumor mass in mice fed a KD diet was also reported in another study, but blood BHB concentrations, blood glucose levels and metastasis were not assessed [17], making the study difficult to interpret. The cause for the reduction in MMTV-PyMT primary tumor growth in response to the KDs that were implemented in these two studies thus remains unclear.

To the best of our knowledge, the studies we report here are the first to demonstrate unequivocally that sustained ketosis does not impact on metastasis formation. Although the impact of KDs on metastasis has been assessed in a number of syngeneic and xenograft models, due to the study design none of these studies have provided conclusive data. In the context of breast cancer, estrogen receptor-positive human breast cancer cells were xenografted into female NOD Scid gamma mice fed with a KD containing a ketogenic ratio of approximately 6:1 that was initiated on the day of tumor cell inoculation. Although the authors report that the number of hepatic metastases was reduced in the KD group, the statistical significance of the results was not assessed [22]. In another study, mice were fed with a KD containing a ketogenic ratio of approximately 5:1 that was initiated 4 weeks before 4T1 tumor cell inoculation. Similar to our findings, primary tumor growth was temporarily reduced but at the endpoint no significant differences between KD and standard diet-fed mice were observed [20]. Furthermore, the number of circulating tumor cells and the number of pulmonary metastases were assessed, and found to be significantly reduced upon KD. However, the interpretation of these data is limited, as neither BHB nor glucose concentrations in the blood were monitored. In this context it is notable that our data suggest that a KD with a ratio of 5:1 is not sufficient to induce sustained ketosis in BALB/c mice. Moreover, the temporary reduction in primary tumor growth in response to the KD could have resulted in reduced metastatic seeding, which was also not controlled for in this study. The effect of KDs on metastasis has also been addressed in a study in which glioblastoma cells were implanted subcutaneously in the abdominal region of experimental mice. Metastasis was tendentially but not statistically significantly reduced [37]. However, the model used is highly artificial, metastasis was only indirectly assessed, primary tumor size fluctuated, the ketogenic ratio was not mentioned, and blood BHB levels did not even reach 0.5 mM, all limiting the value of the data significantly.

Our observations have important ramifications for the design and interpretation of studies with KDs in mice. While BHB concentrations above 0.5 mM reflect physiological nutritional ketosis [38], medical guidelines recommend BHB concentrations between 2 and 5 mM for therapeutic purposes [1]. In patients, this therapeutic level of ketosis is usually achieved with a ketogenic ratio of 4:1. Here we report that in contrast to the situation in humans, the ketogenic ratios needed to be much higher in BALB/c and MMTV-PyMT mice in order to achieve such BHB concentrations in the blood. Consistently, this phenomenon has been noted earlier also for CD-1^nu/nu^ mice [39]. Although the authors of this study did not provide any corresponding data, they state: “As mice are able to keep blood glucose levels and show lower ketosis on 2:1 to 4:1 diets, compared to humans, we decided to use an 8:1 diet in our mouse model, to reach at least a ketosis over 2 mmol/L” [39].

For experiments in which-tumor bearing mice were fed KDs, varying ketogenic ratios have been used, ranging from 2.7:1 (e.g. [40]) to 9:1 (e.g. [27]). The mean BHB concentrations determined in the blood of mice in tumor experiments varied between about 0.8 [41] and 4.5 mM [42]. Notably, the ketogenic ratio of KDs given to experimental mice does not always correspond with the BHB concentrations actually induced by the diet. For example, while a KD with a ketogenic ratio of 6:1 induced mean BHB concentrations of 4.5 mM in NOD SCID mice [42], the same ketogenic ratio induced a maximum BHB concentration of 1 mM in mice with a C57/Bl6 background [41]. In contrast, in HsdCpb:NMRI-Foxn1^nu^ mice, BHB concentrations of about 1.5 mM were reached with a relatively low ketogenic ratio of just 3:1 [40]. Although these differences suggest the existence of strain-specific effects of the ketogenic ratio on blood BHB levels, a study in which C57BL/6J, FVB/NJ, A/J, and DBA/2J mice were fed a KD with a ketogenic ratio of 4.3:1 showed that all four mouse strains uniformly reached mean blood BHB concentrations of between 1 and 1.4 mM, with no significant differences between the strains [43]. However, the authors did not assess BHB concentrations longitudinally, but only once, namely 2 weeks after KD initiation [43]. Our observations suggest that BHB concentrations fluctuate and can temporarily peak around 2 mM, even when KDs with lower ketogenic ratios are fed, and sustained ketosis is not achieved. The degree to which strain-to-strain differences in ketosis induction exist in response to KDs therefore remains to be fully investigated.

In the study mentioned above, in which the impact of a KD on ketosis in mice with C57BL/6J, FVB/NJ, A/J, and DBA/2J genetic backgrounds was compared, only male mice were used [43]. This may have further influenced the outcome of this study, since we report here that male mice did not respond to KDs containing ketogenic ratios up to 1:10 with a sustained increase in blood BHB concentrations. This finding is supported by a study in which the effect of a KD with a ketogenic ratio of 6:1 on cachectic lung tumor-bearing Kras^G12D/+^; Lkb1^f/f^ mice was assessed [44]. While female mice had a mean BHB concentration of 1.8 mM, male mice only reached a statistically significantly lower mean of 1.3 mM [44]. The BHB concentration of 1.3 mM was above the mean basal BHB levels of approximately 0.3 mM in male Kras^G12D/+^; Lkb1^f/f^ mice, suggesting low-level ketosis [44]. However, we observed temporarily increased and partially fluctuating BHB concentrations in some male animals (see Fig. 1a), indicating that the timepoint at which BHB concentrations are assessed has an impact on the results. This fact further emphasizes the necessity to assess BHB levels longitudinally throughout an experiment.

In summary, our findings suggest that ketogenic ratios need to be tested and established for individual mouse strains when performing experiments with KDs. Moreover, BHB levels should be monitored longitudinally throughout the experiment, and not only at single timepoints that may not be representative. In addition, the sex -specific differences in ketosis induction in mice has to be taken into consideration.

The original rationale behind the use of KDs in the context of cancer was the notion that KDs are characterized by a low carbohydrate intake, and should lead to a reduction of glucose in the blood (for a review see [13]). Since tumors are considered to be dependent on glycolysis, a phenomenon described by the well-known Warburg effect [45], it was reasoned that KDs should slow down tumor cell proliferation and subsequently tumor growth. In fact, the reduction of EMT6 [18] and MMTV-PyMT [19] primary tumor growth upon KDs with a ketogenic ratio of 4:1 discussed above was accompanied by a significant reduction in blood glucose levels [18, 19] that may have contributed to the observed reduction of tumor growth, despite the suboptimal induction of ketosis. However, a number of observations suggest that KDs can impact tumor growth independently of changes in blood glucose levels. For example, several preclinical studies with gastric and colon carcinoma reported that primary tumor growth was reduced in KD-fed mice, despite unchanged blood glucose levels (e.g. [40, 46]). We also observed a temporarily reduced primary tumor growth in a spontaneous metastasis assay with 4T1 cells despite unchanged glucose levels in the blood. Furthermore, we did not observe impaired primary MMTV-PyMT tumor growth despite a significant reduction of blood glucose concentrations. Mechanisms other than blood glucose reduction must therefore contribute to the growth inhibitory effects of KDs on tumors.

It has recently been suggested that activation of the BHB receptor Hcar-2 and the subsequent induction of the *Hopx* gene that encodes the homeodomain-only protein may be a mechanism that fosters growth inhibitory effects of KDs on tumors [27]. Consistent with this notion, the homeodomain-only protein can influence the Wnt-pathway [47–49] that is also active in mammary tumors (reviewed in [50]), and has been shown to play an important role in the growth and metastasis of MMTV-PyMT tumors [51]. However, we report here that Hcar-2 was ubiquitously expressed in both MMTV-PyMT and 4T1 tumors, but that Hcar-2 expression was not significantly affected by KD. Therefore, it is likely that additional as yet unidentified mechanisms exist.

Together, our data suggest that KDs can temporarily reduce tumor growth, but do not affect metastasis in murine triple-negative and luminal breast cancer models, even when sustained BHB concentrations above 2 mM are induced. These data therefore provide preclinical evidence that KDs should not prove detrimental to the outcome of human breast cancer patients. Nevertheless, our study has a number of limitations. We show that induction of ketosis in mice requires KDs with higher ketogenic ratios compared to the case in humans. Furthermore, in contrast to the situation in humans, only female mice developed sustained ketosis in response to the KDs used in our study. Thus, there may be limitations in the transferability of our findings to human patients. Moreover, we used a single cell line-based model for triple-negative breast cancer, and a single autochthonous model for luminal breast cancer, which possibility represents a further limitation in terms of a general applicability of our findings to all triple negative and luminal breast cancers. Despite these limitations, the careful and exhaustive longitudinal control of ketosis and blood sugar levels in the experiments reported here provides a firm foundation for further studies to extend our observations using additional models.

In conclusion, our findings highlight the necessity when designing KD studies in mice to test and establish the ideal ketogenic ratio for individual mouse strains, to monitor BHB levels longitudinally and to consider sex-dependent differences in ketosis. These findings also indicate that conclusions about the impact of KDs on tumor growth and metastasis that are present in the literature may need to be reconsidered, given that a state of sustained ketosis was not achieved in all studies published, and that blood BHB concentrations were usually not assessed longitudinally throughout the duration of the KD, limiting the conclusions that can be drawn from these experiments. Most importantly, our data indicate that KDs have no influence on metastasis in the models investigated even when sustained ketosis is induced, and do not promote primary mammary tumor growth. This suggests that concerns over the safety of these diets in terms of the promotion of tumor growth and progression may be non-founded, meaning that cancer patients should be able to benefit from the improved well-being and quality of life that has been associated with KDs.

### Supplementary Information

Below is the link to the electronic supplementary material.

Supplementary material 1 (DOCX 2105.8 kb)

## References

[1] Gesellschaft für Neuropädiatrie e.V. (GNP) (2023): S1-Leitlinie Ketogene Diäten, 4.1, 30.11.2021. https://register.awmf.org/de/leitlinien/detail/022-021. Accessed 16 Oct 2023

[2] Flowers L, Broder MW, Forsyth C (2003) Toxicological review of acetone (CAS No. 67-64-1). In Support of summary information on the integrated risk information system (IRIS). U.S. Environmental Protection Agency Washington, DC. EPA/635/R-03/004 https://www.epa.gov/iri

[3] Glew RH (2010). You can get there from here: acetone, anionic ketones and even-carbon fatty acids can provide substrates for gluconeogenesis. Niger J Physiol Sci.

[4] Kossoff EH, Wang HS (2013). Dietary therapies for epilepsy. Biomed J.

[5] Fearon KC, Borland W, Preston T, Tisdale MJ, Shenkin A, Calman KC (1988). Cancer cachexia: influence of systemic ketosis on substrate levels and nitrogen metabolism. Am J Clin Nutr.

[6] Klement RJ, Sweeney RA (2016). Impact of a ketogenic diet intervention during radiotherapy on body composition: II. Protocol of a randomised phase I study (KETOCOMP). Clin Nutr ESPEN.

[7] Kämmerer U, Klement RJ, Joos FT, Sütterlin M, Reuss-Borst M (2021). Low Carb and ketogenic diets increase quality of life, physical performance, body composition, and metabolic health of women with breast cancer. Nutrients.

[8] Klement RJ, Sweeney RA (2022). Impact of a ketogenic diet intervention during radiotherapy on body composition: V. final results of the KETOCOMP study for head and neck cancer patients. Strahlenther Onkol.

[9] Nebeling LC, Miraldi F, Shurin SB, Lerner E (1995). Effects of a ketogenic diet on tumor metabolism and nutritional status in pediatric oncology patients: two case reports. J Am Coll Nutr.

[10] Schmidt M, Pfetzer N, Schwab M, Strauss I, Kämmerer U (2011). Effects of a ketogenic diet on the quality of life in 16 patients with advanced cancer: a pilot trial. Nutr Metab.

[11] Cohen CW, Fontaine KR, Arend RC, Soleymani T, Gower BA (2018). Favorable effects of a ketogenic diet on physical function, perceived energy, and food cravings in women with ovarian or endometrial cancer: a randomized, controlled trial. Nutrients.

[12] Klement RJ (2017). Beneficial effects of ketogenic diets for cancer patients: a realist review with focus on evidence and confirmation. Med Oncol.

[13] Klement RJ (2018). Wilhelm Brünings’ forgotten contribution to the metabolic treatment of cancer utilizing hypoglycemia and a very low carbohydrate (ketogenic) diet. J Tradit Complement Med.

[14] Klement RJ, Brehm N, Sweeney RA (2020). Ketogenic diets in medical oncology: a systematic review with focus on clinical outcomes. Med Oncol.

[15] Bonuccelli G, Tsirigos A, Whitaker-Menezes D, Pavlides S, Pestell RG, Chiavarina B, Frank PG, Flomenberg N, Howell A, Martinez-Outschoorn UE, Sotgia F, Lisanti MP (2010). Ketones and lactate fuel tumor growth and metastasis: evidence that epithelial cancer cells use oxidative mitochondrial metabolism. Cell Cycle.

[16] Martinez-Outschoorn UE, Lin Z, Whitaker-Menezes D, Howell A, Sotgia F, Lisanti MP (2012). Ketone body utilization drives Tumor growth and Metastasis. Cell Cycle.

[17] Gluschnaider U, Hertz R, Ohayon S, Smeir E, Smets M, Pikarsky E, Bar-Tana J (2014). Long-chain fatty acid analogues suppress breast tumorigenesis and progression. Cancer Res.

[18] Talib WH (2020). A ketogenic diet combined with melatonin overcomes cisplatin and vincristine drug resistance in breast carcinoma syngraft. Nutrition.

[19] Zou Y, Fineberg S, Pearlman A, Feinman RD, Fine EJ (2020). The effect of a ketogenic diet and synergy with rapamycin in a mouse model of Breast cancer. PLoS ONE.

[20] Wang X, Liu X, Jia Z, Zhang Y, Wang S, Zhang H (2021). Evaluation of the effects of different dietary patterns on breast cancer: monitoring circulating tumor cells. Foods.

[21] Weber DD, Aminzadeh-Gohari S, Tulipan J, Catalano L, Feichtinger RG, Kofler B (2020). Ketogenic diet in the treatment of cancer—where do we stand?. Mol Metab.

[22] Zuo Q, Mogol AN, Liu YJ, Santaliz Casiano A, Chien C, Drnevich J, Imir OB, Kulkoyluoglu-Cotul E, Park NH, Shapiro DJ, Park BH, Ziegler Y, Katzenellenbogen BS, Aranda E, O’Neill JD, Raghavendra AS, Tripathy D, Madak Erdogan Z (2022). Targeting metabolic adaptations in the breast cancer-liver metastatic niche using dietary approaches to improve endocrine therapy efficacy. Mol Cancer Res.

[23] Guy CT, Cardiff RD, Muller WJ (1992). Induction of mammary tumors by expression of polyomavirus middle T oncogene: a transgenic mouse model for metastatic disease. Mol Cell Biol.

[24] Lin EY, Jones JG, Li P, Zhu L, Whitney KD, Muller WJ, Pollard JW (2003). Progression to malignancy in the polyoma middle T oncoprotein mouse breast cancer model provides a reliable model for human diseases. Am J Pathol.

[25] Mähler Convenor M, Berard M, Feinstein R, Gallagher A, Illgen-Wilcke B, Pritchett-Corning K, Raspa M, FELASA working group on revision of guidelines for health monitoring of rodents and rabbits (2014). FELASA recommendations for the health monitoring of mouse, rat, hamster, guinea pig and rabbit colonies in breeding and experimental units. Lab Anim.

[26] Klement RJ, Champ CE, Otto C, Kämmerer U (2016). Anti-tumor effects of ketogenic diets in mice: a meta-analysis. PLoS ONE.

[27] Dmitrieva-Posocco O, Wong AC, Lundgren P, Golos AM, Descamps HC, Dohnalová L, Cramer Z, Tian Y, Yueh B, Eskiocak O, Egervari G, Lan Y, Liu J, Fan J, Kim J, Madhu B, Schneider KM, Khoziainova S, Andreeva N, Wang Q, Li N, Furth EE, Bailis W, Kelsen JR, Hamilton KE, Kaestner KH, Berger SL, Epstein JA, Jain R, Li M, Beyaz S, Lengner CJ, Katona BW, Grivennikov SI, Thaiss CA, Levy M (2022). β-Hydroxybutyrate suppresses colorectal cancer. Nature.

[28] Fine EJ, Segal-Isaacson CJ, Feinman RD, Herszkopf S, Romano MC, Tomuta N, Bontempo AF, Negassa A, Sparano JA (2012). Targeting insulin inhibition as a metabolic therapy in advanced cancer: a pilot safety and feasibility dietary trial in 10 patients. Nutrition.

[29] Branca JJ, Pacini S, Ruggiero M (2015). Effects of pre-surgical vitamin D supplementation and ketogenic diet in a patient with recurrent breast cancer. Anticancer Res.

[30] Jansen N, Walach H (2016). The development of tumours under a ketogenic diet in association with the novel tumour marker TKTL1: a case series in general practice. Oncol Lett.

[31] Schwalb M, Taubmann M, Hines S, Reinwald H, Ruggiero M (2016). Clinical Observation of a Novel, complementary, immunotherapeutic Approach based on Ketogenic Diet, Chondroitin Sulfate, vitamin D3, oleic acid and a fermented milk and Colostrum product. Am J Immunol.

[32] Khodabakhshi A, Akbari ME, Mirzaei HR, Mehrad-Majd H, Kalamian M, Davoodi SH (2020). Feasibility, Safety, and Beneficial effects of MCT-Based ketogenic Diet for Breast Cancer Treatment: a Randomized Controlled Trial Study. Nutr Cancer.

[33] Khodabakhshi A, Seyfried TN, Kalamian M, Beheshti M, Davoodi SH (2020). Does a ketogenic diet have beneficial effects on quality of life, physical activity or biomarkers in patients with Breast cancer: a randomized controlled clinical trial. Nutr J.

[34] Khodabakhshi A, Akbari ME, Mirzaei HR, Seyfried TN, Kalamian M, Davoodi SH (2021). Effects of ketogenic metabolic therapy on patients with Breast cancer: a randomized controlled clinical trial. Clin Nutr.

[35] Rodrigues LM, Uribe-Lewis S, Madhu B, Honess DJ, Stubbs M, Griffiths JR (2017). The action of β-hydroxybutyrate on the growth, metabolism and global histone H3 acetylation of spontaneous mouse mammary tumours: evidence of a β-hydroxybutyrate paradox. Cancer Metab.

[36] Zhuang Y, Chan DK, Haugrud AB, Miskimins WK (2014). Mechanisms by which low glucose enhances the cytotoxicity of metformin to cancer cells both in vitro and in vivo. PLoS ONE.

[37] Poff AM, Ward N, Seyfried TN, Arnold P, D’Agostino DP (2015). Non-toxic metabolic management of Metastatic Cancer in VM mice: Novel Combination of Ketogenic Diet, Ketone Supplementation, and hyperbaric oxygen therapy. PLoS ONE.

[38] Volek JS, Phinney SD (2012). The art and science of low carbohydrate performance.

[39] Licha D, Vidali S, Aminzadeh-Gohari S, Alka O, Breitkreuz L, Kohlbacher O, Reischl RJ, Feichtinger RG, Kofler B, Huber CG (2019). Untargeted Metabolomics reveals Molecular effects of Ketogenic Diet on Healthy and Tumor Xenograft Mouse models. Int J Mol Sci.

[40] Otto C, Kaemmerer U, Illert B, Muehling B, Pfetzer N, Wittig R, Voelker HU, Thiede A, Coy JF (2008). Growth of human gastric cancer cells in nude mice is delayed by a ketogenic diet supplemented with omega-3 fatty acids and medium-chain triglycerides. BMC Cancer.

[41] Stafford P, Abdelwahab MG, Kim DY, Preul MC, Rho JM, Scheck AC (2010). The ketogenic diet reverses gene expression patterns and reduces reactive oxygen species levels when used as an adjuvant therapy for glioma. Nutr Metab.

[42] Dang MT, Wehrli S, Dang CV, Curran T (2015). The ketogenic Diet does not affect growth of hedgehog pathway Medulloblastoma in mice. PLoS ONE.

[43] Dutton SB, Escayg A (2008). Genetic influences on ketogenic diet efficacy. Epilepsia.

[44] Langer HT, Ramsamooj S, Liang RJ, Grover R, Hwang SK, Goncalves MD (2022). Systemic ketone replacement does not improve survival or Cancer Cachexia in mice with Lung Cancer. Front Oncol.

[45] Warburg O, Posener K, Negelein E (1924). Über den stoffwechsel der carcinomzelle. Biochem Z.

[46] Hao GW, Chen YS, He DM, Wang HY, Wu GH, Zhang B (2015). Growth of human colon Cancer cells in nude mice is delayed by ketogenic diet with or without omega-3 fatty acids and medium-chain triglycerides. Asian Pac J Cancer Prev.

[47] Jain R, Li D, Gupta M, Manderfield LJ, Ifkovits JL, Wang Q, Liu F, Liu Y, Poleshko A, Padmanabhan A, Raum JC, Li L, Morrisey EE, Lu MM, Won KJ, Epstein JA (2015). Heart development. Integration of Bmp and wnt signaling by Hopx specifies commitment of cardiomyoblasts. Science.

[48] Palpant NJ, Wang Y, Hadland B, Zaunbrecher RJ, Redd M, Jones D, Pabon L, Jain R, Epstein J, Ruzzo WL, Zheng Y, Bernstein I, Margolin A, Murry CE (2017). Chromatin and Transcriptional Analysis of Mesoderm Progenitor Cells Identifies HOPX as a Regulator of primitive hematopoiesis. Cell Rep.

[49] Liang H, Wang C, Gao K, Li J, Jia R (2019). ΜicroRNA-421 promotes the progression of nonsmall cell lung cancer by targeting HOPX and regulating the Wnt/βcatenin signaling pathway. Mol Med Rep.

[50] Xu X, Zhang M, Xu F, Jiang S (2020). Wnt signaling in Breast cancer: biological mechanisms, challenges and opportunities. Mol Cancer.

[51] Buechel D, Sugiyama N, Rubinstein N, Saxena M, Kalathur RKR, Lüönd F, Vafaizadeh V, Valenta T, Hausmann G, Cantù C, Basler K, Christofori G (2021). Parsing β-catenin’s cell adhesion and wnt signaling functions in malignant mammary Tumor progression. Proc Natl Acad Sci USA.

