# Supplementary material for: Ketogenic diet does not promote triple-negative and luminal mammary tumor growth and metastasis in experimental mice
Source: Clin Exp Metastasis. 2023 Dec 9;41(3):251–66. doi: 10.1007/s10585-023-10249-z (PMC11213782; doi:10.1007/s10585-023-10249-z)
Supplement: Supplementary file 1 — Supplementary material 1 (DOCX 2105.8 kb) [file 10585_2023_10249_MOESM1_ESM.docx]

**Supplementary Information**

**Ketogenic diet does not promote triple-negative and luminal mammary tumor growth and metastasis in experimental mice**

Meret Grube ^1^, Arno Dimmler ^2^, Anja Schmaus ^1^, Rafael Saup ^1^, Tabea Wagner ^1^, Boyan K. Garvalov ^1^, Jonathan P. Sleeman ^1, 3^, Wilko Thiele ^1*^

^1^ Department of Microvascular Biology and Pathobiology, European Center for Angioscience (ECAS), Medical Faculty Mannheim, University of Heidelberg, Mannheim, Germany

^2^ Institute of Pathology, Vincentius Kliniken Karlsruhe, Karlsruhe, Germany

^3^ Institute for Biological and Chemical Systems, Karlsruhe Institute of Technology (KIT), Campus North, Karlsruhe, Germany

**Supplementary Figures**

**
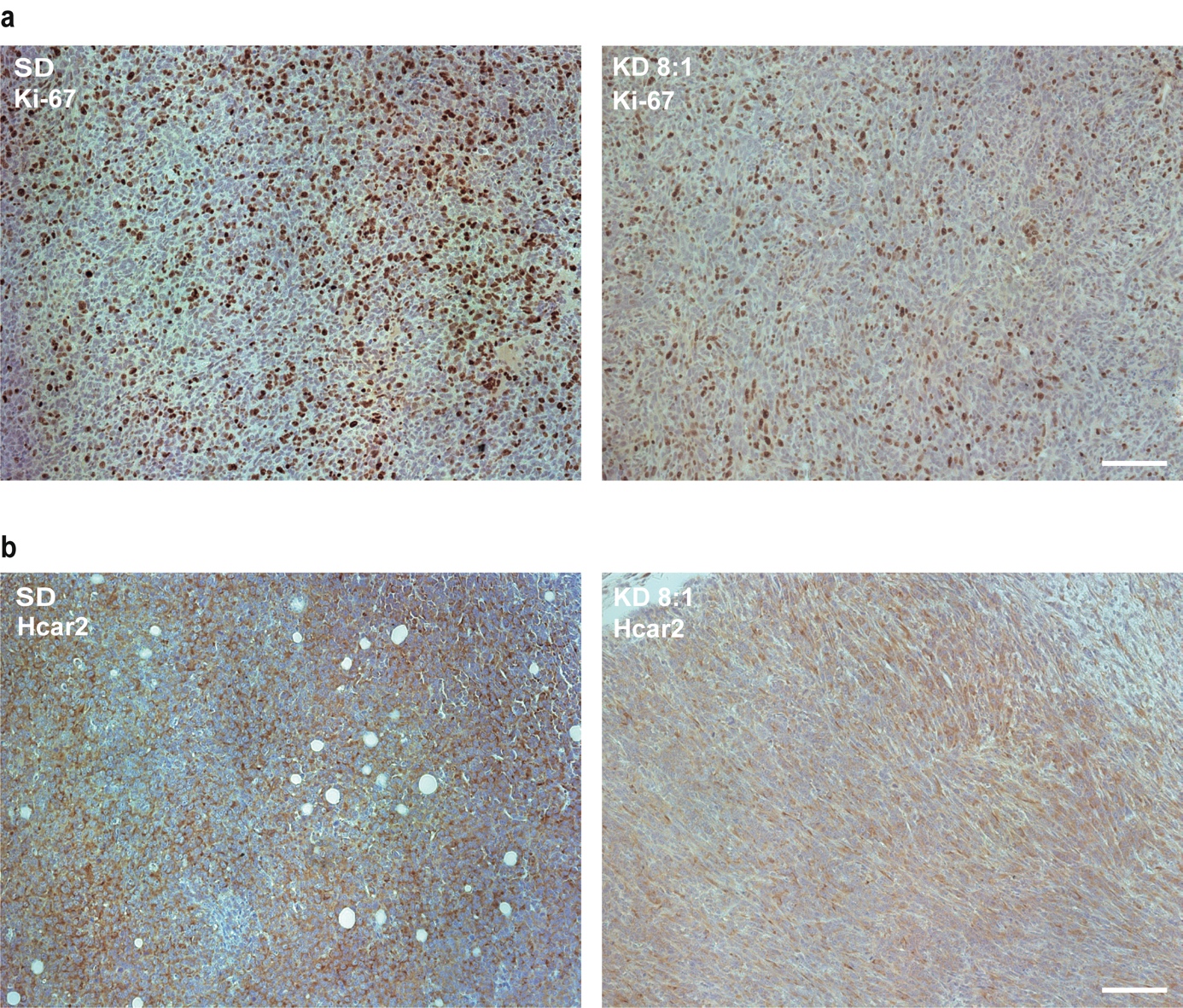
**

**Fig. S1** KD reduces the proliferative index, but does not affect Hcar2 expression of tumor cells within primary tumors in a spontaneous metastasis assay upon orthotopic implantation of 4T1 mammary carcinoma cells. Female BALB/c mice were either fed standard diet (SD; N =11 animals) or ketogenic diet (KD; N = 6 animals) with a ketogenic ratio of 8:1 *ad libitum*. 4T1 cells (1×10^6^ each animal) were injected orthotopically into the mammary fat pads of syngeneic BALB/c mice 10 days after the initiation of KD. Paraffin sections of the primary tumors were stained with antibodies specific for Ki-67 (**a**) or Hcar2 (**b**). (**a**) Overview images of the Ki-67 stained tumor sections from animals fed with SD or KD, acquired at low magnification (see Fig. 2f for higher magnification images). (**b**) Overview images of the Hcar2 stained tumor sections from animals fed with SD or KD, acquired at low magnification (see Fig. 2i for higher magnification images). Scale bars: 100 μm.


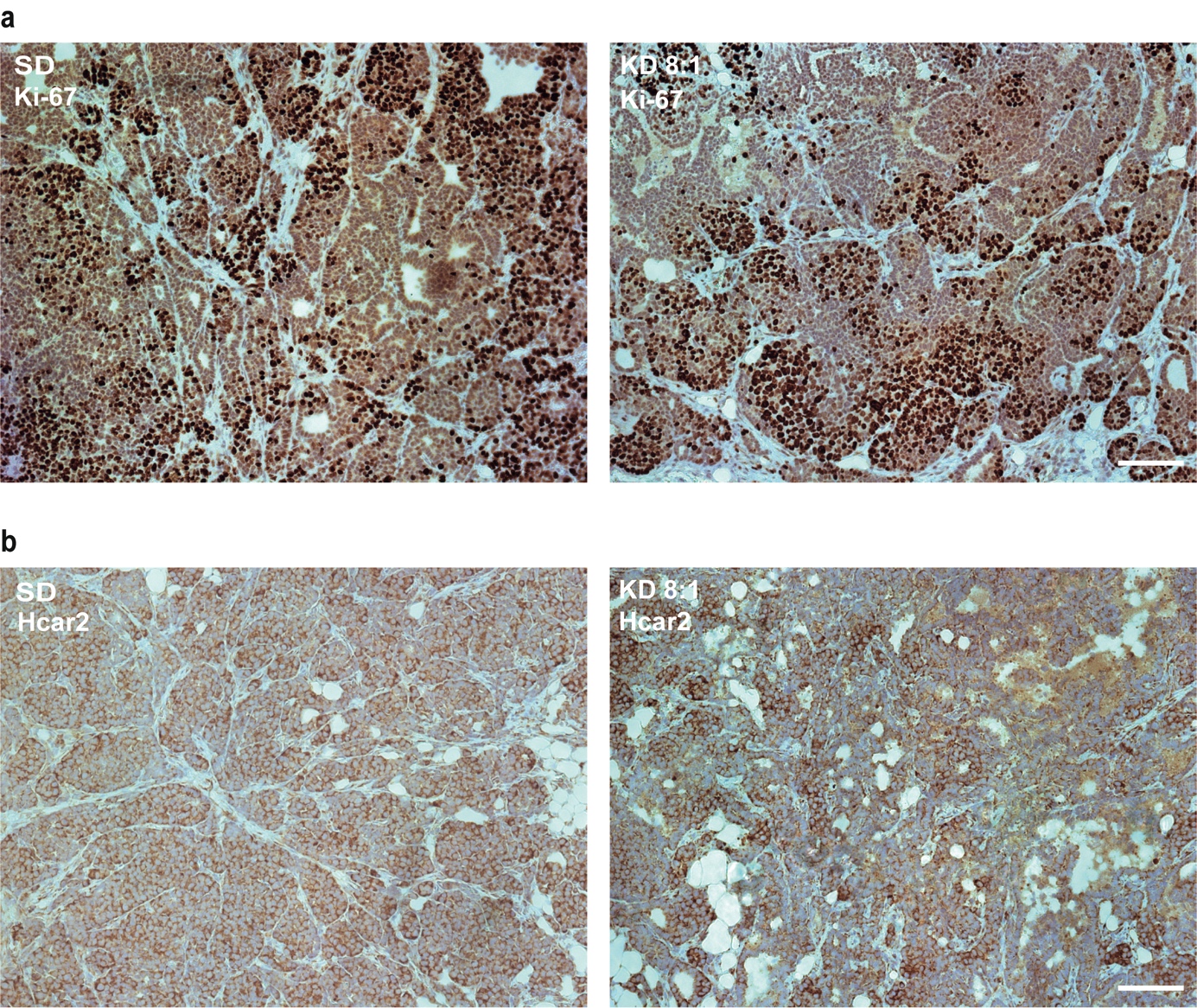


**Fig. S2** KD does not affect the proliferative index or Hcar2 expression of autochthonous luminal MMTV-PyMT tumors. MMTV-PyMT mice (N = 13 females per group) aged 8 weeks were either fed standard diet (SD) or ketogenic diet (KD) with a ketogenic ratio of 8:1 *ad libitum*. Paraffin sections of the primary tumors were stained with antibodies specific for Ki-67 (**a**) or Hcar2 (**b**). (**a**) Overview images of the Ki-67 stained tumor sections from animals fed with SD or KD, acquired at low magnification (see Fig. 5f for higher magnification images). (**b**) Overview images of the Hcar2 stained tumor sections from animals fed with SD or KD, acquired at low magnification (see Fig. 5i for higher magnification images). Scale bars: 100 μm.
